# Factors that influence utilisation of HIV/AIDS prevention methods among university students residing at a selected university campus

**DOI:** 10.1080/17290376.2014.986517

**Published:** 2014-12-02

**Authors:** Eléazar Ndabarora, Gugu Mchunu

**Affiliations:** ^a^ MN, is a student of Master in Community Health Nursing, University of KwaZulu-Natal, Durban, South Africa; ^b^ Ph.D., is an Associate Professor and Head of Nursing Discipline at the School of Nursing and Public Health, University of KwaZulu-Natal, Durban, South Africa

**Keywords:** HIV/AIDS prevention methods, utilisation, university students, méthodes de prévention du VIH/SIDA, utilisation, étudiants universitaires

## Abstract

Various studies have reported that university students, who are mostly young people, rarely use existing HIV/AIDS preventive methods. Although studies have shown that young university students have a high degree of knowledge about HIV/AIDS and HIV modes of transmission, they are still not utilising the existing HIV prevention methods and still engage in risky sexual practices favourable to HIV. Some variables, such as awareness of existing HIV/AIDS prevention methods, have been associated with utilisation of such methods. The study aimed to explore factors that influence use of existing HIV/AIDS prevention methods among university students residing in a selected campus, using the Health Belief Model (HBM) as a theoretical framework. A quantitative research approach and an exploratory-descriptive design were used to describe perceived factors that influence utilisation by university students of HIV/AIDS prevention methods. A total of 335 students completed online and manual questionnaires. Study findings showed that the factors which influenced utilisation of HIV/AIDS prevention methods were mainly determined by awareness of the existing university-based HIV/AIDS prevention strategies. Most utilised prevention methods were voluntary counselling and testing services and free condoms. Perceived susceptibility and perceived threat of HIV/AIDS score was also found to correlate with HIV risk index score. Perceived susceptibility and perceived threat of HIV/AIDS showed correlation with self-efficacy on condoms and their utilisation. Most HBM variables were not predictors of utilisation of HIV/AIDS prevention methods among students. Intervention aiming to improve the utilisation of HIV/AIDS prevention methods among students at the selected university should focus on removing identified barriers, promoting HIV/AIDS prevention services and providing appropriate resources to implement such programmes.

## Résumé

Diverses études ont rapporté que les étudiants universitaires, qui sont pour la plupart jeunes, utilisent rarement les méthodes de prévention du VIH/SIDA à leur porté. Bien que les études ont montré que les jeunes étudiants universitaires ont un niveau élevé de connaissances du VIH/SIDA ainsi que les modes de transmission du VIH, ces étudiants n'utilisent pas ces méthodes de prévention du VIH et se livrent encore à des pratiques sexuelles risquant et favorables au VIH. Certaines variables, telles que la sensibilisation aux méthodes de prévention du VIH/SIDA existants à l'université, ont été associées à l'utilisation de ces méthodes. Cette étude avait l'objectif d’étudier les facteurs qui influencent l'utilisation des méthodes de prévention du VIH/SIDA qui sont disponibles aux étudiants universitaires résidant dans un campus sélectionné, en utilisant le modèle de croyance à la santé (HBM) comme un cadre théorique. L'approche quantitative et la conception exploratoire-descriptive ont été utilisés pour décrire les facteurs qui influencent l'utilisation des méthodes de prévention du VIH/SIDA tels que perçus par les étudiants universitaires. Un total de 335 étudiants ont rempli des questionnaires électroniquement et manuellement. Les résultats de cette étude ont montré que les facteurs qui ont influencé l'utilisation des méthodes de prévention du VIH/SIDA étaient principalement déterminés par la sensibilisation des stratégies de prévention du VIH/SIDA disponibles à l'université. Les méthodes de prévention les plus utilisées étaient les services de conseil et dépistage volontaires et les préservatifs gratuits. Le score de susceptibilité et de menace du VIH/SIDA perçues a montré une corrélation avec le score de l'indice de risque du VIH.La susceptibilité et la menace du VIH/SIDA perçues a montré de corrélation avec l'auto-efficacité sur les préservatifs et leur utilisation. La plupart des variables du modèle de croyance à la santé (HBM) n’étaient pas des prédicteurs de l'utilisation des méthodes de prévention du VIH/SIDA parmi les étudiants.Intervention visant à améliorer l'utilisation des méthodes de prévention du VIH/SIDA parmi les étudiants de l'université choisie devrait se concentrer sur l'élimination des obstacles identifiés, la promotion des services de prévention du VIH/SIDA, et fournir des ressources appropriées pour implémenter ces programmes.

## 1. Introduction

UNAIDS/WHO (2008) reported that by the end of 2007, approximately 33.2 million people worldwide were living with HIV, and 2.5 million people were newly infected with HIV – 45% of these being young people aged between 14 and 25 years. The report emphasised that preventing new HIV infections remains the most powerful weapon to fight and reverse the epidemic, especially among young people. Sub-Saharan Africa remains the most heavily affected region of the world, accounting for approximately two-thirds of all incident and prevalent HIV infections and three-quarters of all AIDS deaths (Shisana, Rehle, Simbayi, Zuma, Jooste, Pillay-van-Wyk, *et al*. 2009; Vitoria, Granich, Gilks, Gunneberg, Hosseini, Were, *et al*. 2009).

In South Africa, the national HIV/AIDS prevalence rate at the end of 2007 was estimated to be 18.1% (UNAIDS/WHO 2008). Young people between 15 and 24 years of age account for over five million new HIV infections worldwide each year, with an estimated 6000 youth becoming infected each day across the globe (UNAIDS 2006). The findings of a survey conducted in South Africa by Pettifor, Straten, Dunbar, Shiboski and Padian (2004) showed that young South Africans aged 15–24 years had a high HIV prevalence of 10.2%; of these, 77% were women.

Studies conducted in African countries such as Ghana, Nigeria and Kenya have identified university students as a group at high risk for HIV infection due to their risky sexual behaviours (Adam & Mutungi 2007; Mberia & Mukulu 2011; Oppong & Oti-Boadi 2013; Osonwa, Eko, Abeshi & Offiong 2013). Various studies have reported on female university students engaging in cross-generational sex (Ntata, Muula, Siziya & Kayambazinthu 2008) where female students have sex with older men who are able to offer financial compensation in the form of school fees, clothing, food and cell phones. Cross-generational sex seems to be a substantial source of infection among South African youth, with one generational population presenting with higher and the other with lower HIV prevalence (Katz & Low-Beer 2008). Such relationships expose female students to risky sexual behaviours. Nor are young women the only ones engaging in risky sexual behaviours. Recent studies have shown that 68% of South African young women and 56% of young men under 25 years of age were engaging in high-risk sexual behaviours (Simbayi, Kalichman, Jooste, Mfecane & Cain 2005).

In response to the HIV/AIDS pandemic, HIV/AIDS policies and HIV prevention programmes have been put in place in all institutions of higher learning in South Africa and most have implemented interventions aimed at preventing HIV/AIDS (Van Wyk & Pieterse 2006). These interventions comprise existing HIV/AIDS prevention policy, education and awareness raising – especially the ABC message (Abstain, Be faithful, use a Condom) – voluntary counselling and testing (VCT) for HIV, affordable antiretroviral therapy (ART), HIV/AIDS wellness programmes, peer education programmes and support services, and integration of HIV/AIDS issues in teaching, research and service activities (Van Wyk & Pieterse 2006). Reviewed literature shows that HIV prevention methods at institutions of higher learning have mainly focused on knowledge, awareness and practices (Mberia & Mukulu 2011; Reddy & Frantz 2011), but Reddy and Frantz argue that high level of knowledge about HIV/AIDS is not in itself sufficient to promote behaviour change among South African university students.

To increase utilisation of HIV prevention strategies, some institutions of higher learning have made condoms freely available, extensively promoting their use and helping to overcome social and personal obstacles that limit their use (Mberia & Mukulu 2011; Policy Project 2000; William & Cherly 1999). A study on HIV/AIDS risk factors among students conducted at the University of the Free State by Badenhorst, van Staden and Coetsee (2008) showed that there was improved utilisation of HIV/AIDS prevention methods by university students and that students were highly aware that they were at high risk of contracting HIV infection. While these HIV prevention strategies have been widely advocated as potentially effective in the fight against the AIDS pandemic, it has nevertheless emerged in various studies that the sexual behaviour of young university students does not correspond with their heightened awareness and knowledge about HIV/AIDS and HIV modes of transmission (Ntata *et al*. 2008; Simbayi *et al*. 2005), as they continued to engage in unprotected sexual behaviour that put them at high risk of contracting HIV infection (Badenhorst *et al*. 2008; Hartell 2005; Kaiser Family Foundation 2006). These findings demonstrate that existing HIV prevention methods continue to be underutilised by university students.

There is therefore a need to determine what factors influence the utilisation of existing HIV/AIDS prevention methods, and what the barriers are to their utilisation (as perceived by students) so that relevant measures can be put in place.

## 2. Purpose of the study

The purpose of this study was to explore factors that influence the use of existing HIV/AIDS prevention methods among university students residing at a selected campus.

## 3. Methodology

The study was guided by the Health Belief Model (HBM), as developed in the 1950s by a group of psychologists (Hochbaum, Rosenstock, and Kegels) working in the US Public Health Services (Glanz, Rimer & Viswanath 2008:46). The model attempts to describe health-seeking behaviours (Rosenstock 1974) and has been widely used both to explain change and maintenance of health-related behaviours and as a guiding framework for health behaviour interventions. The HBM suggests that health-related action depends upon (a) the existence of sufficient motivation or health concern to make health issues relevant (*Perceived Susceptibility*) (b) the belief that one is susceptible to a serious health problem or to sequelae of that illness (*Perceived Threat*) and (c) the belief that following a particular health recommendation would be beneficial in reducing the perceived threat (*Perceived Severity*), at a subjectively acceptable cost (Rosenstock, Strecher & Becker 1988). The model hypothesises that for an individual to take action, he must decide that the behaviour creates a serious health problem, and that he is personally susceptible to its health harm, and that moderating or stopping the behaviour will be beneficial.

For instance, in HIV/AIDS-related studies, the theory may seek to explain why sexually active youth indulge in risky sexual behaviours. The guiding components of the model may explain that youth who have low risk perception of being infected with HIV or sexually transmitted infection (STI) (*Perceived Susceptibility*) are likely to engage in risky sexual behaviour, and that such behaviour will expose them to serious health problems such as HIV or STIs, unwanted pregnancy or other sexual and reproductive health problems (*Perceived Severity*). The youth who decide to use condom correctly and consistently will always be protected from these unwanted health problems and consequently live a healthy life (*Perceived Benefit*). In previous studies, the HBM has concluded that the youth who were adherent to consistent condom usage had higher perceived susceptibility scores and lower perceived barrier as compared to those who did not intend to use HIV preventive measures, whose perceived benefits were lower. Perceived susceptibility is necessary before commitment to change these risky behaviours can occur (Glanz *et al*. 2008).

A quantitative research approach was followed to explore variables and examine relationships among them. An exploratory–descriptive design was used to describe the perceived factors that influence the utilisation by university students of HIV/AIDS prevention methods.

## 4. Research setting

The study was conducted at on- and off-campus residences of a selected campus at the University of KwaZulu-Natal (UKZN), South Africa.

## 5. Population, sampling and sample size

The university residences of selected Durban campuses accommodate approximately 2162 students. The on-campus residences are grouped into two halls; one of these halls comprises 12 individual residences, and the other has two components: a cluster residence and a six-storey residence. There are also two off-campus residences, giving a total of 14 residences. Students residing in selected halls of these residences were invited to participate in the study.

Simple random sampling was used to select three on-campus residences, and purposive sampling was used to select one off-campus residence. Purposive sampling was chosen because there were only two off-campus residences, and including them in the sampling frame for random sampling would reduce their chance of being selected in the sample. Respondents were selected using convenience sampling. In each residence, students who were available at the time of data collection were invited to participate in the study. Online questionnaires were linked to a created web link whereby the questionnaires were sent back by respondents after completion.

## 6. Data collection procedure

The researcher approached respondents in their respective residences in the evenings. Students who consented to participate in the study completed the questionnaires manually. A ballot box where respondents deposited their completed questionnaires was provided. A similar questionnaire was sent and completed by respondents online. This questionnaire incorporated a link whereby completed questionnaires could be sent back.

## 7. Research instrument description

A pre-existing questionnaire by Simbayi *et al*. (2005) on ‘Risk Factors for HIV/AIDS among Youth in Cape Town, South Africa’ was used. The questionnaire was adapted to make it relevant to the current study.

The questionnaire comprised six sections in which questions were designed to collect data relating to the following categories: (a) socio-demographic data, (b) perceived susceptibility, perceived threat and attitude towards HIV/AIDS, (c) knowledge of HIV/AIDS and sources of information, (d) sexual experiences and risky sexual behaviour, (e) awareness of on-campus HIV prevention methods and their utilisation and (f) perceived barriers, perceived benefits and self-efficacy in relation to utilisation of HIV/AIDS prevention methods.

Additional questions were added to the original questionnaire drawn up by Simbayi *et al*. (2005), and the research was conducted in a different setting from the earlier study. A pilot study was therefore conducted to test design issues.

The online pilot questionnaire was sent by the researcher to five students and another five students were given the questionnaires manually to complete. Findings of the pilot study showed that the questionnaire was easy to understand and contained no ambiguities or misunderstandings.

## 8. Data analysis

Data from both online and manual questionnaires were analysed using the Statistics Package of Social Sciences (SPSS) Version 15–0.

### 8.1. Descriptive statistics

Categorical variables were summarised using frequency distributions (counts and percentages) in tables, bar charts and graphs. Numerical variables were summarised by measures of central tendency; mean, mode and median; and measures of variability.

### 8.2. Analytic statistics

The chi-square test was used to further describe relationships, similarities and differences between categorical independent variables and dependent variables, and to establish demographic differences among respondents. Correlations were performed to assess relationships between quantitative variables. Because most compared variables did not show statistically significant differences, the multivariate regression to assess many independent variables simultaneously was not performed.

## 9. Ethical consideration

The research proposal was approved by the Ethics Committee of the University. Permission to conduct the study was also obtained from the Dean of Students and the Housing Administrator. Informed consent was duly sought from respondents. For the online questionnaire, consent was obtained by the fact that respondents accepted to complete the questionnaire, and this was clarified in the Information document. Assurance and adherence to confidentiality of elicited information were maintained throughout the study. Participation in the study was voluntary. Respondents were informed that they could stop or withdraw from participating in the study at any time without any penalty.

## 10. Results

Three hundred and sixty questionnaires were distributed among students who reside in 4 residences in a selected campus, out of which 261 (72.5%) returned the completed questionnaires. With the online questionnaires, completed and returned questionnaires were received from 74 respondents. There were thus a total of 335 respondents, which represented 15.5% of the whole population.

Students were asked a series of questions relating to variables such as awareness of existing HIV/AIDS prevention methods on their campus and university-wide, knowledge of HIV/AIDS and encouraging factors and perceived barriers to utilising these methods. Furthermore, they were asked questions relating to their utilisation of these existing methods.

### 10.1. Socio-demographic characteristics of respondents

The age of the participants ranged from 17 to 48 years with a mean age of 22.9 years, a median of 22 years and a mode of 21 years. Other demographic information is presented in Table 1.

**Table 1.** Socio-demographic data of respondents.

Socio-demographic attributes (*n *= 335)	%
Gender	
Female	52.8
Male	47.2
Marital status	
Single	78.2
Married	7.5
Widow	0.9
Girl-/boyfriend	13.1
Divorced	0.3
Ethnicity/race	
African/black	92.2
Indian	2.4
White	4.2
Coloured	1.2
Student status	
Local students	78.2
International	21.8
Academic level	
Undergraduate	83
Postgraduate	17

### 10.2. Awareness of HIV/AIDS prevention methods

**Fig. 1.**
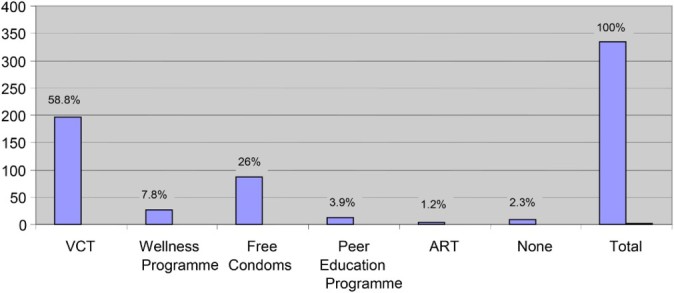
Awareness of HIV/AIDS prevention methods.

Just over half of respondents were aware of VCT, 197 (58.8%); of free condoms, 87 (26%); and of the Wellness Programme, 26 (7.8%). Only 13 (3.9%) were aware of the Peer Education Programme and just 4 (1.2%) were aware of ART. Eight (2.3%) respondents did not report awareness of any HIV/AIDS prevention methods (Table 2 and Fig. 1).

**Table 2.** Awareness and utilisation of HIV/AIDS prevention methods.

HIV/AIDS prevention methods	Awareness (%) (*n *= 335)	Utilisation (%) (*n *= 335)
VCT	58.8	37.9
Wellness programme	7.8	4.78
Free condoms	26	43.58
Peer education programme	3.9	2.09
ART	1.2	0.61
None	2.3	11.04

### 10.3. Utilisation of on-campus HIV/AIDS prevention methods

**Fig. 2.**
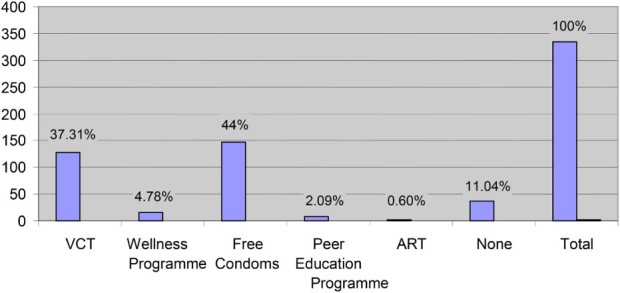
Utilisation of HIV prevention methods.

Even though a majority of students indicated awareness of VCT as an HIV/AIDS prevention method, only 37.9% of students had utilised this service, while 43.9% had utilised free condoms, making this the most utilised on-campus HIV/AIDS prevention method. The least utilised preventive methods were the Wellness Programme, by 4.8% of respondents; the Peer Education Programme, by 2%; and ART by just two respondents (0.6%) (Table 2 and Fig. 2).

#### 10.3.1. ABC methods

Respondents were asked to report on their utilisation of ABC as a prevention method, on- and off-campus. One hundred and sixty-two (49.5%) respondents reported that they used condoms, 86 (26.3%) reported that they abstained and 79 (24.2%) reported that they were faithful.

#### 10.3.2. HIV testing and other HIV/AIDS prevention methods

Altogether, more than two-thirds, 249 (76.4%) respondents had been tested for HIV, while 76 (23%) had never been tested for HIV. A large majority, totalling 283 (86.3%) respondents, reported that they planned to be tested and 45 (13.7%) reported that they did not plan to be tested.

### 10.4. Relationship between identified variables and utilisation of HIV prevention methods

There was no relationship between any socio-demographic variables and utilisation of HIV/AIDS prevention methods.

#### 10.4.1. Awareness

A chi-square test to compare awareness and utilisation of HIV/AIDS prevention methods showed a statistically significant difference between awareness and utilisation; the *χ* ^2^ value was 5.838, with *p*-value .047. There was a strong association between awareness and HIV/AIDS prevention methods utilisation. The degree of agreement between these two variables was also high (98.3%) (Table 3).

**Table 3.** Association between awareness and utilisation of HIV/AIDS prevention methods.

		Awareness of HIV/AIDS prevention methods		Total	Pearson's *χ*^2^ value	*p*-Value at 95% CI
		No	Yes			
Utilised campus HIV/AIDS prevention methods	No	3	34	37	5.838	0.047
		8.1%	91.9%	100%		
	Yes	5	293	298		
		1.70%	98.3%	100%		
Total		8	327	335		
		2.4%	97.6%	100%		

### 10.5. Relationships between demographic variables and HIV testing

Fifty-six per cent of female respondents had been tested for HIV, as compared to 43.9% of males. There was, however, no statistically significant difference between HIV testing and gender; the *χ* ^2^ value was 3.367 and the *p*-value was .052 at 95% CI.

In relation to condom usage, 50.8% of females compared with 49.2% of males had used condoms in the most recent sexual intercourse. There was also no gender difference on condom usage: the *χ* ^2^ value was 1.623; the *p*-value was .203 at 95% CI.

### 10.6. Perceived barriers, benefits and self-efficacy, and HIV/AIDS prevention methods utilisation

#### 10.6.1. Perceived barriers and self-efficacy on abstinence

A majority, totalling 286 (86.4%) of respondents, were in agreement that abstinence was a way to protect oneself from getting HIV infection, while 59 (37.1%) agreed that abstinence was not practical (Table 2). The perceived barriers and self-efficacy minimum score value was 1, the maximum was 20 and the mean was moderately low to high at 12.06 (60.3%).

#### 10.6.2. Perceived susceptibility/perceived threat and self-efficacy on abstinence

The perceived susceptibility and perceived threat mean score was 21.3881 against 12.0604 abstinence score. The Pearson correlation value was 0.057, at 95% confidence interval. There was therefore no correlation between perceived susceptibility/perceived threat and self-efficacy on abstinence.

#### 10.6.3. Perceptions and self-efficacy on condom usage

Fourteen (4.4%) and 30 (9.5%) respondents, strongly agreed, or agreed, respectively, that they did not use condoms because they trusted their sexual partner. Forty-three (13.7%) and equally 43 (13.7%) respondents agreed strongly, or agreed, that condoms were unnatural. In addition, 6 (2%) and 25 (8.2%) respondents strongly agreed, or agreed, that their sexual partners did not like condoms. Thirteen (4.2%) and 59 (18.9%) respondents strongly agreed, or agreed, that condoms decrease sexual pleasure, and 29 (9.4%) and 20 (6.5%) respondents strongly agreed, or agreed, that they were not able to negotiate condom usage with their sexual partner. A substantial majority of 197 (60.8%) and 74 (22.8%) respondents strongly agreed, or agreed, that condoms were easy to get, but 62 (19.4%) and 77 (24.1%) strongly agreed, or agreed, that condoms were sometimes not available (Table 4).

**Table 4.** Perceived barriers, perceived benefits and self-efficacy.

Statements	Strongly agree (%)	Agree (%)	Neutral (%)	Disagree (%)	Strongly disagree (%)	Sample size (*n*)
*Abstinence*						
Abstaining is a way of protecting oneself from getting HIV	69.8	16.6	4.8	3.6	5.1	331
Abstinence is not practical for me	18.2	17.9	22.7	16.1	25.2	330
Being faithful to one uninfected partner protects from getting HIV	32.1	20.5	21.7	11.9	13.8	327
It is difficult to be faithful to my partners	4.7	8.8	11.6	29.8	45.1	319
*Condom use*						
I don't use a condom because I trust my partner	4.4	9.5	18.1	23.8	44.1	315
Condoms are unnatural	13.7	13.7	21.1	21.7	29.7	313
My partner doesn't like to use a condom	6.2	8.2	17.7	30.2	37.7	305
Using condom decreases sexual pleasure	4.2	18.9	22.8	24	30.1	312
I am not able to negotiate with my partner to use of a condom	9.4	6.5	15	25.4	43.6	307
Condoms are easy to get	60.8	22.8	4.9	4.3	7.1	324
Sometimes condoms are not available	19.4	24.1	13.8	17.2	25.6	320

#### 10.6.4. Relationships between perceived susceptibility and perceived threat score and self-efficacy on condom use

The mean score for perceived susceptibility and perceived threat was 21.3881 against 23.4667 for perceptions score on condom use. A Pearson correlation to test the relationship between perceived susceptibility/perceived threat of HIV/AIDS and self-efficacy on condom use was 0.80 at 95% confidence interval. There was therefore correlation between perceived susceptibility and perceived threat of HIV/AIDS and self-efficacy on condom and condom use (Table 4).

#### 10.6.5. Relationship between perceptions and self-efficacy on VCT services, condoms and their utilisation

Mean difference between condom perceptions/self-efficacy score and condom usage was −0.725054; *p*-value was .539 at 95% confidence interval [CI = −3.02576 to 1.58469]. In addition, mean difference between VCT perceptions/self-efficacy score and HIV testing was −0.62331; *p*-value was .537 at 95% confidence interval [CI = −2.60903 to 1.36241]. There was therefore no relationship between these perceptions and utilisation of HIV/AIDS prevention methods.

### 10.7. Factors encouraging the use of HIV/AIDS preventive methods

The most-reported factors encouraging HIV preventive method utilisation were student movements against HIV/AIDS, reported by 19 (5.9%); inclusion of HIV/AIDS programme in academic curriculum, reported by 19 (5.9%); and having open days against HIV/AIDS, reported by 15 (4.7%).

### 10.8. Perceived barriers to the use of HIV/AIDS prevention methods

The most reported barriers were chiefly issues around VCT and awareness, as summarised in Tables 5 and 6.

**Table 5.** Reported barriers to the use of HIV/AIDS prevention methods among students.

Reported barriers to the use of HIV/AIDS preventive measures	% (*n* = 225)
Lack of privacy of the HIV testing venue	19.6
Uninsured confidentiality	9.3
Long appointment for HIV testing	8
Lack of awareness of HIV services	31.6
Feeling uncomfortably towards peers as they know you	15.6
Poor counselling	3.6
Peers are too young	4
Unfriendly clinic staff	3.1
Long lines when HIV testing	5.3

**Table 6.** Reported barriers to the use of HIV/AIDS prevention methods among students (*n *= 225).

Reported barriers to the use of HIV/AIDS preventive measure	Frequency	%
Lack of privacy of the HIV testing venue	44	19.6
Uninsured confidentiality	21	9.3
Long appointment for HIV testing	18	8
Lack of awareness of HIV services	71	31.6
Feeling uncomfortably towards peers as they know you	35	15.6
Poor counselling	8	3.6
Peers are too young	9	4
Unfriendly clinic staff	7	3.1
Long lines when HIV testing	12	5.3

## 11. Discussion of results

The findings of this study showed that student demographics such as age and gender were not predictors of utilisation of HIV/AIDS prevention methods. However, though there were no significant statistical differences among these variables, results showed that Indian respondents used on-campus VCT more than black students, in the order of 5 (71.4%) against 120 (43.6%). It was also noted that White respondents made no use of on-campus VCT but they all used condoms, 100% (12), whereas less than half of black respondents, 130 (47.3%), used condoms and only 28.6% of Indian respondents used condoms. These findings suggest very low condom use among black and Indian respondents, which is a concern for HIV/AIDS prevention among university students. Also, there was no significant difference between undergraduate and postgraduate students in utilisation of HIV/AIDS prevention methods.

The findings of the current study showed further that the overall mean of perceived susceptibility and perceived threat of HIV/AIDS was high (76.4%), with standard deviation (SD = 3.185). In addition, 76.5% of respondents were in agreement that the possibility of become infected with HIV/AIDS was a matter of concern for them. However, their high perceived susceptibility to HIV/AIDS did not correlate with use of any HIV/AIDS prevention methods. This finding differed from the findings of previous studies carried out in the former University of Durban-Westville which revealed that students did not perceive themselves as susceptible to contracting HIV/AIDS and that their use of those prevention methods was very low (Stremlau & Nkosi 2001; Uys, Alexander, Martin & Ichharam 2001).

While similar studies conducted in Nepal (Iriyama, Nakahara, Jimba, Ichikawa & Wakai 2007) and in Kenya (Othero, Aduma & Opil 2009) showed that students did perceive themselves as susceptible to HIV infection as a consequence of their previous sexual experience, these studies nonetheless showed troublingly low utilisation of existing on-campus HIV/AIDS prevention methods.

The findings of the current study also showed that even though the overall mean knowledge of HIV/AIDS score among students was high, at 82.22%, students nonetheless harboured significant misconceptions about HIV modes of transmission; a very large majority of 209 (92.8%) students reported that one cannot get HIV by having sex with a virgin, and a mere 8 (2.4%) respondents reported that they did not know how one could contract HIV. As predicted in previous studies, knowledge of HIV/AIDS in this study did not correlate with utilisation of any of the HIV/AIDS prevention methods. These findings correspond with earlier studies conducted in South Africa, China and Kenya which reported that university students had gaps in knowledge of HIV mode of transmission that needed to be addressed through health education (Lonn, Sahlholm, Maimaiti, Abdukarim & Anderson 2007; Othero *et al*. 2009; Stremlau & Nkosi 2001; Uys *et al*. 2001). This finding suggests that even though there are strategies in place that focus on HIV/AIDS awareness and information giving, there is still an urgent need to address issues relating in particular to HIV/AIDS modes of transmission that are a potential concern for young people. Although the previous studies have reported that students' awareness and high level of knowledge about existing HIV/AIDS prevention methods did not correlate with their utilisation (Ergene, Cok, Tumer & Unal 2005; Fagen & Flay 2009), the current study showed positive results in that the prevention methods which students reported awareness of were also those most utilised – two examples being free condoms, 146 (43.58%) and VCT, 127 (37.91%). As expected, the findings showed that students were unlikely to utilise programmes that they were not aware of. The least utilised preventive methods were the Wellness Programme, 16 (4.78%); the Peer Education Programme, 7 (2.09%); and ART, 2 (0.6%). Only 31.2% perceived peer educators to be helpful, and 44.5% felt that the programme was not helpful. This was a disappointing finding, as one would expect wellness and peer education programmes to be at the forefront in providing necessary information regarding HIV/AIDS prevention.

Identified barriers to HIV testing included fear of being stigmatised, fear of testing positive, low self-efficacy and low perceived susceptibility. Similar barriers were identified in the study conducted in India by Chakrapani, Shanmugam, Michael, Velayudham and Newman (2008) on factors influencing HIV testing. Also identified as barriers were: location of HIV testing centres closer to residences; waiting time for counselling, testing and getting test results; lack of same-day HIV testing methods; and incompetent and insensitive counsellors.

## 12. Conclusion and recommendations

It emerged from the findings of this study that most HBM variables were not predictors of HIV/AIDS prevention methods utilisation among university students. Interventions for HIV/AIDS prevention among students at UKZN should consequently focus on removing identified barriers by providing a favourable HIV testing venue that would ensure confidentiality, employing the same-day HIV testing method, advertising HIV/AIDS prevention services, providing correct knowledge of HIV/AIDS (especially in relation to HIV modes of transmission) and focusing on behavioural change.

Follow-up studies should explore the appropriateness and comprehensiveness of the existing HIV prevention methods, including wellness programmes, in order to determine why they are not being utilised.

A specific recommendation would be to include HIV/AIDS information in the academic curriculum so as to offer comprehensive and correct information on HIV/AIDS to students. Also needed is a comparative follow-up study, involving several countries, to explore measures and best practices to improve utilisation of HIV/AIDS prevention methods.
